# Genomic and Pathogenicity Mechanisms of the Main *Theobroma cacao* L. Eukaryotic Pathogens: A Systematic Review

**DOI:** 10.3390/microorganisms11061567

**Published:** 2023-06-13

**Authors:** Diogo Pereira Silva de Novais, Thiago Mafra Batista, Eduardo Almeida Costa, Carlos Priminho Pirovani

**Affiliations:** 1 Department of Biological Sciences, Center for Biotechnology and Genetics, State University of Santa Cruz (UESC), Ilhéus 45662-900, BA, Brazil; 2 Bahia Federal Institute of Education, Science and Technology (IFBA), Porto Seguro 45810-000, BA, Brazil; 3 Environmental Science Training Center, Federal University of Southern Bahia (UFSB), Porto Seguro 45810-000, BA, Brazil

**Keywords:** *Moniliophthora*, *Phytophthora*, *Ceratocystis cacaofunesta*, witches’ broom disease, black pod disease, Ceratocystis wilt, frosty pod rot

## Abstract

A set of diseases caused by fungi and oomycetes are responsible for large losses in annual world cocoa production. Managing the impact caused by these diseases is very complex because a common solution has yet to be found for different pathogens. In this context, the systematic knowledge of *Theobroma cacao* L. pathogens’ molecular characteristics may help researchers understand the possibilities and limitations of cocoa disease management strategies. This work systematically organized and summarized the main findings of omics studies of *T. cacao* eukaryotic pathogens, focusing on the plant–pathogen interaction and production dynamics. Using the PRISMA protocol and a semiautomated process, we selected papers from the Scopus and Web of Science databases and collected data from the selected papers. From the initial 3169 studies, 149 were selected. The first author’s affiliations were mostly from two countries, Brazil (55%) and the USA (22%). The most frequent genera were *Moniliophthora* (105 studies), *Phytophthora* (59 studies) and *Ceratocystis* (13 studies). The systematic review database includes papers reporting the whole-genome sequence from six cocoa pathogens and evidence of some necrosis-inducing-like proteins, which are common in *T. cacao* pathogen genomes. This review contributes to the knowledge about *T. cacao* diseases, providing an integrated discussion of *T. cacao* pathogens’ molecular characteristics, common mechanisms of pathogenicity and how this knowledge is produced worldwide.

## 1. Introduction

*Theobroma cacao* L. (Malvaceae) is an important crop for the economy of many countries, as it is used to produce cocoa powder and cocoa butter, important raw materials for the production of foods and cosmetics [1]. *Theobroma cacao* is cultivated mainly in countries in South and Central America as well as in Western and Central Africa, and in some countries in Asia. Despite the large number of producers, cocoa production is continuously challenged by a considerable set of diseases that have a significant impact on the global annual yield.

Diseases caused by microorganisms have been responsible for the main losses of *Theobroma cacao* in different parts of the world over the last decade [2]. Three diseases have received special attention in the scientific literature: witches’ broom disease (WBD) and frosty pod rot (FPR), caused by the basidiomycete fungi *Moniliophthora perniciosa* and *Moniliophthora roreri*, respectively, as well as black pod rot (BPR) caused by *Phytophthora* spp. oomycetes [3,4].

Witches’ broom disease and frosty pod rot (FPR) occur in South and Central America, currently affecting almost all cocoa producers in this region [5]. Black pod root (BPR) occurs in all regions of the world and is caused by different species of *Phytophthora* spp. *Phytophthora palmivora* is distributed globally, but it does not cause the most aggressive forms of BPR. In contrast, *Phytophthora megakarya* only occurs in some countries in West Africa, but it causes the most aggressive losses to trees and yield [6]. In South America, *Phytophthora capcisi* is widespread [7], and *Phytophthora theobromicola* and *P. palmivora* are present in Brazil.

Two other fungi have attracted interest to the scientific community: the basidiomycete *Ceratobasidium theobromae*, which causes vascular-streak dieback (VSD) and currently causes the second-largest yield losses in Southeast Asia, and the ascomycete *Ceratocystis cacaofunesta*, the cause of Ceratocystis wilt disease (CWD), which can kill the host 10–30 days after infection [2].

The scientific community and cocoa farmers have been using different strategies to control *T. cacao* diseases, such as crop management [8], chemical and biological control [9], and the development of resistant cultivars in breeding programs [10].

An important issue is that sometimes a clone resistant to one disease is susceptible to another. For example, the CCN 51 genotype, while being resistant to witches’ broom disease, is susceptible to Ceratocystis wilt disease [11]. Therefore, some researchers have tried to develop genotypes that are simultaneously resistant to more than one disease [12].

In recent decades, some important data about *T. cacao* pathogens’ genomes and molecular aspects of interaction with the host during infection have been produced. The complete genomes of some pathogens, such as *M. perniciosa* [13,14], *M. roreri* [14,15], *Phytophthora* spp. [16,17] and *C. cacaofunesta* [18] have already been sequenced and are publicly available. Moreover, pathogen effectors with important roles in the plant–pathogen interaction are known, such as necrosis and ethylene-inducing proteins (NEPs) from fungi of the genus *Moniliophthora* [19], oomycetes of the genus *Phytophthora* [20], and cerato-platanin proteins (CPs) from the species *M. perniciosa* [21], *M. roreri* [15] and *C. cacaofunesta* [18]. In silico studies have predicted potential effectors for *Moniliophthora* spp. [14] and *C. cacaofunesta* [18] that can be targeted in future research to better understand the molecular aspects of plant–pathogen interactions.

Recent reviews about *T. cacao* diseases describe important aspects of symptomatology, pathogen taxonomy, management strategies and molecular aspects of plant–pathogen interaction. For WBD, Santos et al. [22] presented the evolution of different proteins of *M. perniciosa* in the three phases of infection: the initial secretion of effector proteins to penetrate plant tissues and CPs to overcome the initial plant immune response in the asymptomatic phase; NEPs and proteins associated with catabolic processes such as amylases, pectinases and cellulases in the green broom phase; and the presence of pectin methyl esterase and methanol oxidase proteins in the necrotrophic phase.

In another review, Jiménez et al. [23] reported important genetic aspects of the *M. roreri* population structure, evidencing the presence of more diverse genetic groups in Colombia and other less diverse groups that are more widespread as a consensus of molecular markers studies. This study also presents modulations of fungal gene expression, with a high expression in genes related to cell wall restructuring and the glyoxylate cycle, suggesting that the pathogen perceives and responds to the shortage of nutrients in the late infection period.

For the species of the genera *Phytophthora* which causes cocoa black pod rot, Marelli et al. [24] identified a larger number of genes in *P. megakarya* and *P. palmivora* genomes in comparison to other *Phytophthora* species, which is possibly due to whole-genome duplication events. In the genome of these pathogens, the presence of proteins commonly involved in plant–pathogen interactions, such as pectinases, proteases, elicitins, Crinklers, necrosis-inducing proteins (NLPs) and RXLRs, was observed.

To face the problem of multiple diseases occurring in the same region, it is important to understand potential solutions and limitations considering two or more pathogens simultaneously. Thus, understanding the similarities and specificities of pathogens is essential to propose and evaluate new solutions. In this context, this work aims to summarize the state of the art of molecular studies in eukaryotic cocoa pathogenic microorganisms and what is known about their mechanisms of pathogenicity. To achieve this, we systematically reviewed the available molecular literature about *T. cacao* fungi and oomycete pathogens, especially studies related to plant–pathogen interactions.

## 2. Materials and Methods

This work followed the PRISMA guidelines for reporting systematic reviews [25]. This systematic review included the steps of planning, execution, and data summarization, which were followed by a bioinformatic analysis of the collected data. During the first three steps, we used the StArt software (State of the Art through Systematic Review), Beta version 3.0.3 [26] and R software, version 4.0.3 [27], along with some specific packages mentioned further in this section, to organize the work and provide automation to repetitive tasks.

### 2.1. Planning

The planning started with defining the subject and its main aspects, such as the objective, research questions, paper databases, research string, and data summarization strategy. The planning step was discussed with the research group and reviewed to minimize bias of the protocol. The research questions that guided this review are presented in Table 1.

Terms associated with the research subject were chosen to gather the initial review records. Metadata were collected from primary study papers that contained the terms “cocoa” or “cacao” and “pathogen(s)” or “disease(s)” in the title, abstract or keywords. We limited the study period from 2000 to June 2022 and obtained the works from the Scopus and Web of Science databases. The search strings used in the advanced search in each database are shown in Table 2.

The complete protocol defined in the planning step can be found in Supplementary Materials Table S1.

### 2.2. Execution

Metadata from the studies were collected in June 2022 using advanced search tools in the Scopus and Web of Science, and were downloaded as raw text files in the Bibtex format. Given the large number of initial records obtained from the databases, a semiautomated process based on text mining was used during the screening step. In this process, papers were clustered based on similarities in their abstract content. Papers belonging to clusters not related to the subject of this work were removed from the initial records. The semiautomated record selection consisted of the following steps:

1. The structure of topics present in the records was modeled using latent Dirichlet allocation [28] with the R package textmineR, version 3.0.5 [29]. The number of topics was estimated using the R package ldatuning, version 1.0.2 [30].
2. Records were clustered based on their relation to the topic structure using affinity propagation [31] with the R package APCluster, version 1.14.10 [32]. The number of clusters was estimated with the R package NbClust, version 3.0.1 [33], which provides the most frequent best solution among a set of estimates proposed by different criteria.
3. Word clouds were generated using the R package wordcloud, version 2.6 [34] based on the paper abstracts of each cluster. This allowed for identification and exclusion of clusters not related to the subject of this review. Before excluding a cluster, some papers were randomly selected, and their titles and abstracts were read first.
4. Papers in the selected clusters were subjected to conventional screening using the StArt software.
5. After the semiautomated screening, the titles and abstracts were read, and the remaining papers obtained from the databases were read fully to select those for the next step of analysis.

### 2.3. Summarization

As defined in the review protocol, a dataset was collected from each selected paper. This set consisted of the title, document object identifier (doi), year of publication, genus and species studied, type of molecular analysis, genomic sequences investigated, genes related to pathogenicity or differentially expressed, and proteins differentially accumulated during the plant–pathogen interaction.

A MySQL relational database was constructed to organize the collected data, and the R package RMariaDB, version 1.2.2 [35] was used to connect R with the MySQL database. Graphics were generated using the package ggplot2, version 3.4.0 [36].

In addition to the graphics produced from the summarization of the collected data, metadata from the scientific databases were employed with the R package bibliometrix, version 4.0.1 [37] to generate a co-citation network by coupling the main references. The resulting graph was edited using the Vosviewer software, version 1.6.18 [38].

After this initial data summarization, the genes and proteins linked to the diseases were subjected to bioinformatic analysis.

### 2.4. Bioinformatic Analysis

The amino acid sequences of proteins associated with the diseases were obtained from the NCBI protein database [39] using the identifier provided in the corresponding papers. For genes, the corresponding protein sequences were also obtained from the NCBI database using a translated BLAST search (blastx) [40] to find proteins with high similarity ($e-value<e^{-10}$). A BLAST search was also performed to retrieve protein sequences from other species included in this systematic review.

Clusters of orthologous proteins among the studied genera and species were identified by submitting the protein sequences found to the web version of the Orthovenn2 software [41], which was also used to identify common motifs in the amino acid sequences from different species belonging to the same cluster.

Selected clusters of proteins associated with pathogenicity, shared among pathogenic species, were submitted to Multiple Expectation Maximization for Motif Elicitation using the MEME suite [42,43] to detect shared motifs.

## 3. Results

### 3.1. Bibliometric Analysis

A total of 3617 records were obtained in the initial search, with 1975 records from the Scopus database and 1642 from Web of Science. After removing 1035 records detected as duplicates by the bibliometrix package, the remaining 2852 records were submitted to topic modeling, and subsequently to cluster analysis.

The ldatuning package enabled the estimation of 14 topics as the local optimal number of topics, and we used this number as a parameter for topic modeling. Then, by using the topic modeling result as an input, NbClust also estimated 14 clusters as the local optimal number of clusters, for a total set of 2852 records. After selecting records based on cluster analysis, four clusters containing a total of 841 records were formed comprising studies related to the review subject, and the remaining records were excluded. The excluded clusters were mainly related to human health, benefits of cocoa consumption, agroforestry systems, and other topics not related to the molecular studies of *T. cacao* pathogens. The cluster analysis word clouds for all included and excluded records are in Supplementary Materials Figure S1.

**Figure 1:**
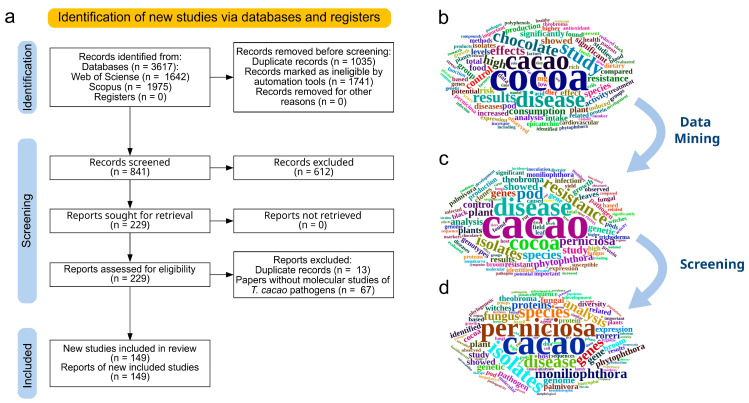
Identification and selection of papers for the systematic review database. (**a**) Flowchart according to PRISMA guidelines. (**b**) Word cloud of abstracts from initial records. (**c**) Word cloud of abstracts after the screening process using data mining. (**d**) Word cloud of abstracts included in the systematic review studies database.

Afterward, the titles and abstracts of the 841 selected records were read, and 229 papers were selected for full reading. Finally, the full reading led to the detection of 13 duplicates, and another 67 papers were excluded because of the absence of molecular studies with *T. cacao* pathogens. The remaining 149 papers formed this systematic review’s database (SRDB) (Figure 1a). In Figure 1b, the initial records had a high frequency of words such as “chocolate”, “food” and “dietary”, all of which were not related to this review subject. The word cloud generated with the records after the semiautomated selection supported by data mining (Figure 1c) no longer contained those words, showing the efficacy of the semi-automated selection. Similarly, the word cloud generated with the abstract of the final selected papers presented a high frequency of words such as “perniciosa”, “moniliophthora”, “fungus”, “phytophthora”, “isolates” and others, demonstrating the efficacy of the screening process (Figure 1d).

**Figure 2:**
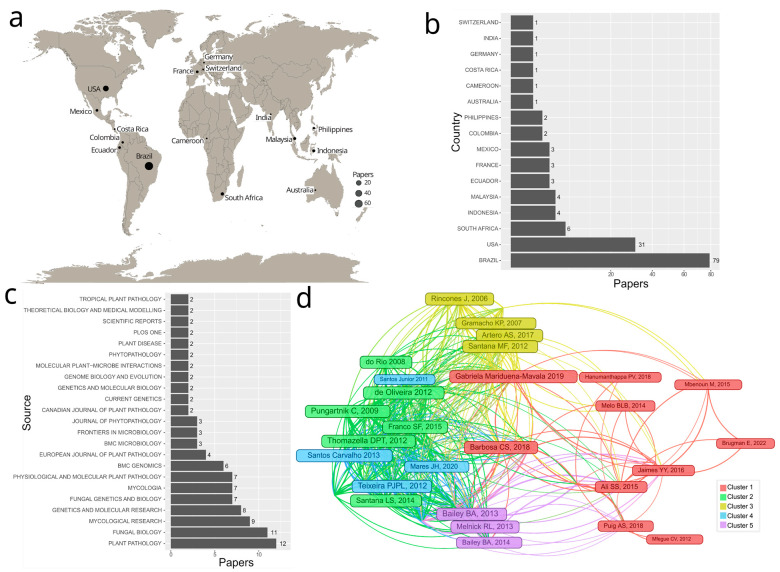
Knowledge production and dissemination about cacao diseases caused by eukaryotic pathogens in the systematic review database. (**a**) Geographic distribution of papers according to the first author’s country. (**b**) Bar plot showing the number of papers by country according to the first author. (**c**) Publication frequency of the most common journals in the systematic review database. (**d**) Co-citation network structure of the most cited papers in the systematic review database. Nodes represent papers and the size of the node represents the number of citations; edges represent the citation between two papers. Nodes with the same color represent clusters of papers with similar co-citation patterns.

The SRDB contained studies conducted by authors from different parts of the world with Eastern Europe being the exception; however, they were mostly authored by researchers from the Americas, and mainly South and Central America (Figure 2a). Almost 80% of the SRDB studies had lead authors from Brazil (55%) and the USA (22%) out of a total of 16 countries (Figure 2b).

The selected studies were predominantly published in journals related to phytopathology and fungal genetics, as expected. Out of 71 journals, approximately 50% of the papers included in this review were published by nine journals: *Plant Pathology*, *Fungal Biology*, *Mycological Research* (currently *Fungal Biology*), *Genetics and Molecular Research*, *Fungal Genetics and Biology*, *Mycologia*, *Physiological and Molecular Plant Pathology*, *BMC Genomics*, and *European Journal of Plant Pathology*.

The most cited papers in the SRDB formed five clusters of co-citations (Figure 2d): cluster 1 contained papers discussing, among other results, the population structure or subpopulation of *M. perniciosa* and *M. roreri* pathogens [14,44,45,46] and *Phytophthora* spp. [47,48]; cluster 2 contained papers discussing *M. perniciosa* effector proteins [49,50,51]; cluster 3 contained papers based on molecular markers discussing *M. perniciosa* genetic diversity, which is similar to cluster 1 [52,53,54]; cluster 4 contained studies characterizing and evaluating *M. perniciosa* proteins interacting with *T. cacao* [55,56,57]; and cluster 5 contained molecular responses of *M. roreri* during its interaction with *T. cacao* [58,59].

### 3.2. Characterization of Studies

**Figure 3:**
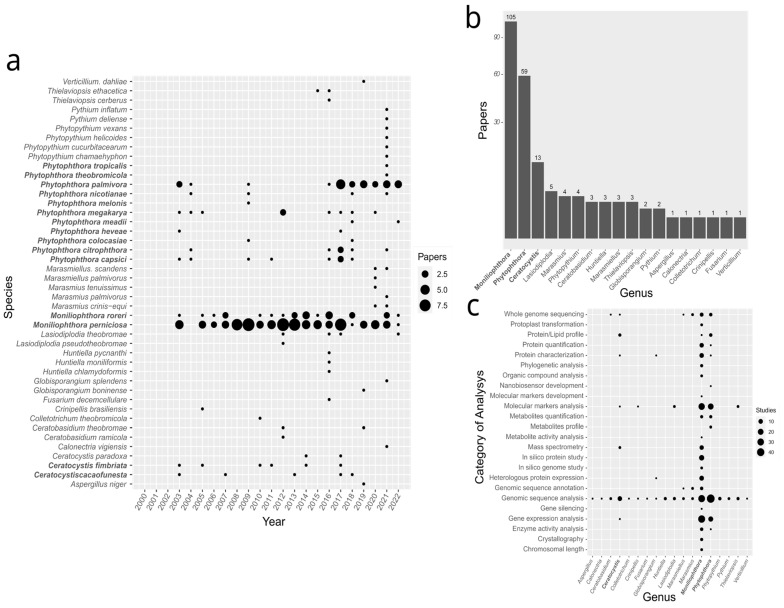
Genus and species of *Theobroma cacao* pathogens and type of analysis. Some papers are counted more than once because they have data from more than one species. (**a**) Number of papers by species per year. (**b**) Bar plot of papers by genus. (**c**) Bar plot of papers by technique for each genus.

The SRDB contained analyses of 44 species distributed across 18 genera. Some species were not exact *T. cacao* pathogens, but were species that received new classifications during or near the time interval of the systematic review. For example, in some papers, samples of *C. cacaofunesta* were analyzed together with *C. fimbriata*, which were collected from different host species. All studies found in the scientific databases were published from 2003 to 2022, although the advanced search was set up to retrieve papers published from the first day of 2000. Papers were concentrated in a set of species, and the yearly distribution indicated an increase from 2017 to 2022 (Figure 3a).

The studies were mostly concentrated on three genera: *Moniliophthora* (105 studies), *Phytophthora* (59 studies) and *Ceratocystis* (13 studies). Within the genera *Moniliophthora* and *Phytophthora*, the most frequent species were *M. perniciosa* and *P. palmivora*, respectively (Figure 3b). It is important to note that the total number of studies represented in any graphic in Figure 3 exceeds the number of papers in the SRDB (149), since some individual papers contain studies of more than one species.

The studies were categorized into 78 techniques according to the type of molecular analysis and/or nature of the results, and those 78 techniques were clustered into 24 categories (Figure 3c). Some techniques were more frequent in the selected studies, such as genomic sequence analysis, molecular marker development and whole-genome sequencing. As expected, the distribution of techniques by genus followed the distribution of studies, concentrating the major number of techniques in the most frequent genera: *Moniliophthora*, *Phytophthora* and *Ceratocystis*, in this order.

The genomic sequence analysis category included studies containing nucleotide sequence analysis for genes from one or more species. This category included studies such as phylogenetic and population structure studies, and specific gene and gene family analysis. Some important studies proposing new species classification were found in this category: (i) the classification of the causal agents of WBD and FPR in a new lineage of *Marasmiceae* [60]; (ii) the classification of *C. cacaofunesta* as a new species in the *C. fimbriata* complex, which has *T. cacao* as a specific host [61]; and (iii) the identification of a new species, *Phytophthora theobromicola*, causing BPR in the Brazilian state of Bahia [62].

The molecular marker analysis category was formed by papers that developed or used existing molecular markers to investigate pathogen genomes. Molecular markers for population studies were developed for *M. perniciosa* [53,63,64], *M. roreri* [46,65] and *Phytophthora* spp. [46,66,67,68].

Some works in the SRDB presented gene expression profiles for pathogens in vitro or those involved in the plant–pathogen interaction. Considering the systematic review protocol, studies only analyzing the host expression were not considered. The SRDB contained studies of gene expression pathogens of the species *M. perniciosa*, *M. roreri*, *P. palmivora* and *P. megakarya.*

The differential gene expression between *M. perniciosa* biotrophic-like and saprotrophic mycelia was first elucidated by Rincones et al. [69] by employing an in vitro culture of isolates from infected *T. cacao* trees. The main results of this study were the identification of the pathogenicity-related genes associated with cell wall degradation as a possible response to carbon, and the description of nitrogen restriction during the biotrophic phase. On the other hand, saprotrophic mycelia genes related to carbon metabolism were overexpressed. Saprotrophic mycelia also overexpressed genes related to antifungal toxins that could prevent the colonization by competing fungi. In another study, Franco et al. [70] identified the presence of Thaumatin-like proteins, a type of pathogenicity-related protein with antifungal effects, in the *M. perniciosa* genome. They observed its expression via in vivo experiments with *T. cacao*.

Another study including the gene expression of *M. perniciosa* identified a family of cerato-platanin (CP) genes that are expressed at different moments during the plant–pathogen interaction. CP proteins usually act as phytotoxins, elicitors and allergens [21].

In samples of *M. roreri* collected from highly susceptible (Pound-7 and CATIE-1000) and tolerant (UF-273, CATIE-R7 and CATIE-R4) clone pods, Bailey et al. [71] demonstrated the earlier expression of genes associated with stress metabolism, responses to heat shock and anoxia in the tolerant clone samples. Genes encoding alternative oxidase proteins and transporter-like genes were among the overexpressed genes in tolerant clone pods, possibly associated with the fungus’s ability to overcome plant resistance. In another experiment, Bailey et al. [72] evaluated the in vitro gene expression of *M. roreri* in the biotrophic phase but obtained results that conflicted with those of in vivo experiments.

Masanto et al. [73] evaluated the relative expression of eight genes associated with pathogenicity from *P. palmivora* isolates obtained from *T. cacao* plants in *Nicotiana benthamiana*. Only four genes were expressed in the in planta test experiment for 24 h, 48 h, 72 h and 96 h. The isolates showed three different levels of severity through in vitro tests with apples, but the mild and severe isolates had similar gene expression profiles, under-expressing the four genes (CNR1, Pec1, Pec3 and RXLR5) in 48 h, while the moderately severe isolate had the opposite behavior.

In another study, Puig et al. [6] evaluated the metabolism and gene expression of *P. palmivora* and *P. megakarya* isolates under high- and low-temperature stress. *P. palmivora* showed more tolerance, which was explained by its persistence in some areas with high-temperature periods, even when plants were also affected by *P. megakarya.* The metabolite analyses showed a higher metabolite concentration in *P. palmivora* than in *P. megakarya* under high-temperature stress. The gene expression profile of genes associated with responses to abiotic stress presented slight differences among isolates. Only genes encoding chaperones/heat shock proteins did not have the same profile: some *P. megakarya* genes were found to be exclusively heat-responsive, while *P. palmivora* genes responded similarly to any type of temperature stress.

Seven papers presented the whole-genome sequencing (WGS) of the *T. cacao* pathogens discussed in this review. The SRDB included WGS and assembly studies for the following species: *C. cacaofunesta* (1); *C. theobromae* (1); *M. perniciosa* (2); *M.* roreri (2); *P. palmivora* (2); and *P. megakarya* (2) (Table 3).

Genome size varies according to genera and species. *Ceratocystis cacaofunesta* and *C. theobromae* have the lowest genome sizes at 30.5 Mb and 31.2 Mb, respectively. *P. megakarya* has the biggest genome size (~222 Mb), followed by *P. palmivora* (~135 Mb), while the genome size of *Moniliophthora* spp. is close to 50 Mb, with there being an exception for the first assembly published by Mondego et al. [13] (26.7 Mb). However, this same work estimated a genome size ranging from 38.7 to 39.0 Mb. The number of predicted genes for each assembly was mostly proportional to the genome size. *C. cacaofunesta* had the smallest number of predicted genes (7382) and *P. megakarya*, the biggest (~57.5 thousand).

Some studies have predicted potentially secreted proteins and/or effector-coding genes for each genome annotation. For *M. perniciosa*, Barbosa et al. [14] predicted 157 effector candidates in the isolate Mp 4145, and more than 100 in the other two isolates from susceptible *T. cacao*. They also predicted 243 effector candidates from the *M. roreri* isolate. Meinhardt et al. [15] identified 1535 secreted protein-coding genes and observed that 1355 among them were expressed in infected cacao pods. Ali et al. [74] identified 138 putative effectors in the *C. theobromae* genome. Ali et al. [16] identified 3757 (1779 transcribed) and 3865 (2633 transcribed) putative, secreted protein-coding genes in the *P. megakarya* and *P. palmivora* genomes, respectively. They also found some of these genes transcribed in RNA samples from infected *T. cacao* plants.

### 3.3. Genes and Proteins Associated with Pathogenicity

In the SRDB, nine papers conducted proteomic studies on proteins associated with pathogenicity or that were differentially accumulated during the plant–pathogen interaction. Almost all (seven) studies analyzed *M. perniciosa* proteins, while one studied *Ganoderma boninense* proteins and another studied *C. cacaofunesta* proteins. However, 17 other studies using molecular markers, gene expression or other nucleotide sequence analysis discussed genes potentially associated with pathogenicity in other species: *M. perniciosa*, *M. roreri*, *P. megakarya* and *P. palmivora*.

Among the *M. perniciosa* protein studies [75], an important work analyzed the fungal proteome from necrotrophic mycelia to basidiocarp development. Some proteins potentially associated with virulence have been found in the primordium (mycelium) and basidiocarp stages, such as aldo-keto reductases, which are associated with virulence and mushroom formation, linoleate diol synthase, leukotriene-A4 hydrolase and 3-ketoacyl-coA-thiolase, and other proteins belonging to biosynthesis pathways of lipids related to immune response and virulence in pathogenic fungi.

In another study, Silva et al. [76] discussed the proteomic response of *M. perniciosa* when exposed to a pathogenesis-related protein TcPR-10 recombinant from a gene isolated from *T. cacao*. Many proteins highly expressed are related to the stress response, such as heat shock proteins and chaperones, and some proteins are associated with defense mechanisms against cytotoxic compounds, such as oxidoreductases and proteins associated with autophagy.

The NLP-like effector of plant necrosis 2 (NEP2), a protein that induces necrosis in the host tissue, plays an important role in *M. perniciosa* infection. Garcia et al. [19] identified NEP1 and NEP2 and demonstrated their ability to induce necrosis by inoculating recombinant NEPs in *Nicotiana tabacum* leaves. The crystal structure of MpNep2 was determined by Zaparoli et al. [77], who also showed that MpNep2 is overexpressed in the stage of advanced necrosis in *T. cacao* tissues.

Other important proteins related to *M. perniciosa* pathogenicity are discussed in other papers in the SRDB. For example, PR-1 proteins were found to neutralize plant defenses and avoid competing fungi in [57]; the crystal structure of MpPR1i was determined by Baroni et al. [78]; and cerato-platanin (CP)-like proteins, another necrosis-inducing protein also found in ascomycetes, were identified by Zaparoli et al. [79].

Genome and secretome analysis of *M. roreri* also revealed the presence and expression of NEPS, PR-like proteins and cerato-platanin proteins [15]. In the same study, Meinhardt et al. [15] found evidence that high expression levels of chitin synthases could help fungi to overcame host defenses.

Teh et al. [80] provided a functional analysis and characterization of *G. boninense* NEPs. The soluble recombinant GbNEP expressed in *Escherichia coli* BL21(DE3)pLysS was able to induce necrosis in two model plants, *Solanum lycopersicum* (tomato) and *Nicotiana tabaccum* (tobacco). However, it was ineffective when applied to oil palm (*Elaeis guineensis*) leaves and root tissues.

Molano et al. [18] identified some proteins potentially associated with pathogenicity secreted by *C. cacaofunesta* in culture media supplemented with *T. cacao* xylem extracts. This set of proteins contains: 25 glycoside hydrolase (GH) proteins associated with cell wall degradation; 2 NEP2 precursors and 1 cerato-platanin protein that are phytotoxic; and 54 phosphatidylinositol (PI)-specific phospholipase Cs (PI-PLCs) among other potential effectors.

NEP-like proteins also play an important role in *Phytophthora* spp. infection. Bae et al. [20] identified multiple copies of the NEP1 protein in the *P. palmivora* genome. From nine copies, six had confirmed expression in the mycelium and one in *P. megakarya* zoospore-infected *T. cacao* leaf tissue. Necrosis-induced proteins are also found in *P. palmivora* genome sequences.

For all genes and proteins presented in the paper as associated with pathogenicity, available amino acid sequences were retrieved from the blastp or blastx search (for nucleotide sequences). A total of 240 retrieved sequences were submitted to Orthovenn2 orthologous cluster analysis.

**Figure 4:**
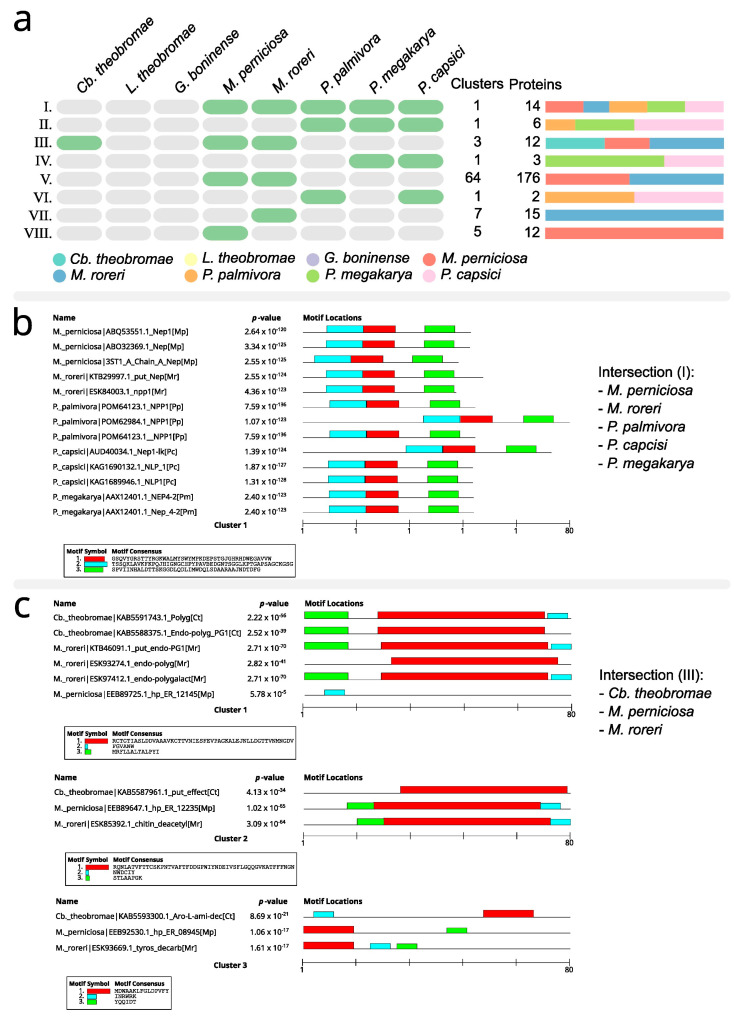
Clusters of orthologous proteins potentially associated with pathogenicity. (**a**) Distribution of clusters by species. (**b**) MEME analysis for a cluster of orthologous proteins from pathogens belonging to the genera *Moniliophthora* and *Phytophthora*. (**c**) MEME analysis for clusters of orthologous proteins from pathogens belonging to the species *M. perniciosa*, *M. roreri* and *Ceratobasidium theobromae*.

Orthovenn2 revealed 83 clusters of possible orthologous proteins among the 240 sequences for different species. Some clusters shown in Figure 4a are formed exclusively by sequences described from pathogens of only one species. These are the clusters in line VII (*M. perniciosa*) and line VIII (*M. roreri*) (Figure 4a). Other clusters are formed by sequences described in pathogens from species belonging to the same genus. These are the clusters in line II (*P. palmivora*, *P. megakarya* and *P. capsici*), line IV (*P. megakarya* and *P. capsici*) and line V (*M. perniciosa* and *M. roreri*). Two set of clusters include sequences from pathogens belonging to different genera: line I (*M. perniciosa*, *M. roreri*, *P. palmivora*, *P. megakarya* and *P. capsici*) and line II (*Ceratobasidium theobromae*, *M. perniciosa* and *M. roreri*). These last two sets of clusters were submitted to MEME analysis for a better description.

Line I (*Moniliophthora* spp. and *Phytophthora* spp.) consisted of a cluster of 14 proteins (Figure 4b). The MEME analysis identified three motifs shared among these proteins.

In line II (*Moniliophthora* spp. and *C. theobromae*), there were three clusters with a total of 12 proteins (Figure 4c). Cluster 1 comprised six proteins with predicted molecular function in cell wall organization, cluster 2 comprised three proteins with predicted molecular function in the carbohydrate metabolic process, and cluster 3 comprised proteins with molecular function predicted in serotonin biosynthetic process from tryptophan.

## 4. Discussion

### 4.1. Identification of Studies and Pathogens

The focus of this systematic review is on the molecular characteristics of *T. cacao* pathogens and diseases. However, given the widespread interest in chocolate, which is the main product produced from the raw material of *T. cacao* pods, it is expected that papers related to the commercial, nutritional and industrial aspects of chocolate will also appear in a scientific database search about *T. cacao*. As a result, the initial database retrieval for this systematic review yielded over 3500 papers. Given this large number of papers, it is useful to have a good strategy for the initial selection of papers.

Recent studies have proposed semiautomated steps carried out by machine learning to assist researchers in their initial search on scientific databases, which face a continuous increase in the amount of data [81,82]. Using a semiautomated strategy saved us time and effort by grouping papers by topics and providing an efficient way to remove papers not related to the systematic review subject. After the initial steps of semiautomated study selection, the total number of papers for title and abstract reading was reduced from 3617 to 817, from which 149 studies were selected for inclusion in the systematic review database (SRDB).

More than half of the papers included in the systematic review have the first author affiliated with a Brazilian institution. This result is consistent with a recent systematic review on the molecular biology of the interaction between cocoa and witches’ broom disease, which showed that Brazilian institutions were responsible for more than 70% of the research on this topic [22].

Cocoa is an important crop for the Brazilian economy, and the country has produced more than 250 thousand tons of cocoa beans in recent years [83]. However, Brazil was the second-largest cocoa producer in the world until the witches’ broom disease spread in the 90s [84]. Since then, much research has been conducted in Brazil, in collaboration with many institutions around the world, on witches’ broom disease and the pathogen *M. perniciosa* [22]. The second largest number of publications in the SRDB are from authors affiliated with American institutions. The global spread of publications does not necessarily reflect the global production of *T. cacao* nor the commercial interesting in the production of cocoa derivates. Despite the interest in the crop, disparities in global scientific production can concentrate a large number of publications in a small number of countries [85]. The United States and Brazil occupied the 1st and 14th positions, respectively, in the SCImago country rankings of publications in 2022 [86], with many collaborations between researchers from multiple institutions regarding *T. cacao* diseases [22]. These two facts together may have contributed to this large number of publications by researchers from Brazilian and American institutions.

In general, the most frequent pathogens analyzed in the SRDB studies have been causing yield losses in cocoa production worldwide in recent years and decades. In the late 1980s, Fulton [3] identified the “trilogy” of diseases caused by fungi and oomycetes in tropical regions, namely, witches’ broom, frost pod rot, and black pod diseases. More recently, the VSD caused by the basidiomycete *C. theobromae* has caused significant losses in cocoa production in Southeast Asia and has been the focus of research [74]. Ceratocystis wilt disease caused by the ascomycete *C. cacaofunesta* [2], which can kill infected plants in 10–30 days [87], has recently been studied more frequently as well. Although being beyond the scope of this review, a complex of badnavirus infecting *T. cacao* trees in the West Africa region, the largest *T. cacao* producer region in the world, is also of concern [88].

The five clusters formed by the most cited papers in the SRDB are mainly related to population structure and the genetic diversity of pathogens. Some important results of these papers have led to the recent identification of new species such as *C. cacaofunesta* [61] and *Phytophthora theobromicola* [62], and the reclassification of *M. perniciosa* and *M. roreri* into a new taxon in the *Marasmiaceae* family [60].

The clear comprehension of pathogen speciation can guide researchers to understand specific characteristics of *T. cacao* pathogens. The whole-genome sequencing of *C. cacaofunesta* showed a phosphatidylinositol-specific phospholipase C (PI-PLC) gene family expansion [18], which is uncommon in other closely related species of the *C. fimbriata* complex. This broad range of PI-PLCs may be related to the pathogens’ ability to overcome susceptible *T. cacao* genotypes’ initial defense [89].

Similar studies have been conducted to understand the genetic diversity of *T. cacao* and to identify molecular markers for disease resistance or tolerance to different diseases such as black pod rot [90,91], witches’ broom disease [92,93,94] and frosty pod rot [95]. Understanding the origin and diversity of cocoa pathogens can help in the development of biocontrol solutions based on coevolved antagonist endophytes [96,97].

### 4.2. Theobroma cacao Pathogens’ Molecular Characterization

Whole-genome sequencing studies were conducted for the most frequent pathogens in this review. *Ceratocystis cacaofunesta* has the smallest genome that is 30.5 Mb and has a few more than 3000 predicted genes, but the range and proportion of the genome size and predicted genes are compatible with other *Ceratocystis* species [18]. The genome size and number of genes from *M. perniciosa* (47.01 Mb and 14.2k genes) and *M. roreri* (47.1 Mb and 14.2k genes) found by Barbosa et al. [14] are consistent with the expected dimensions for these species and with the *M. roreri* genome size found by Meinhardt et al. [15] (52 Mb and 17.9k genes). Slight differences occur even in isolates from different subpopulations or due to different genome sequencing technologies [14]. *Phytophthora palmivora* and *P. megakarya* have the largest genomes and correspondingly the highest number of genes, but these are also consistent with the genus. Ali et al. [16] found genome sizes of 101 Mb for *P. megakarya* and 107 Mb for *P. palmivora*, with ~40k genes. However, Morales et al. [17] found significantly different genome sizes for the same species considering the mean ± SD of the sequenced isolates: 222.04 Mb ± 25.19 (57,577 ± 7904 genes) for *P. megakarya* and 135.32 Mb ± 17.21 (36,778 ± 4481 genes). In *Phytophthora* species, events of hybridization and genome duplication are common [98], and Morales et al. [17] found evidence of recent whole-genome duplications in the genome assemblies. The *C. theobromae* genome has 31.62 Mb (9.2k genes), which is considerably smaller than that of closely related species such as *R. solani* strains (56.02–36.9 Mb) and *Botryobasidium botryosum* (45.75 Mb) [74]. Among the genes and proteins associated with pathogenicity, necrosis-inducing proteins were more frequent in the SRDB studies: *M. perniciosa* [77], *M. roreri* [19], *P. palmivora* and *P. megakarya* [20], and *G. boninense* [80]. Additionally, a NEP2 precursor was also reported in *C. cacaofunesta* [18].

The NEP-like proteins from *Moniliophthora* spp. and *Phytophthora* spp. formed a cluster in the Orthovenn2 analysis, suggesting that they have a similar function. However, the *G. boninense* NEP did not establish an orthologous cluster with the analyzed proteins. This result is consistent with previous findings, which showed that MpNEP is more closely related to oomycetes than fungi, possibly because of gene acquisition for the horizontal transfer by oomycetes from a common ancestor of *M. perniciosa* and *M. roreri* [99]. Moreover, there is evidence of the horizontal acquisition of two other genes by *Moniliophthora*, mannitol phosphate dehydrogenase (MPDH) from actinobacteria and metallo-dependent hydrolase (MDH) from firmicutes [99], which enforces the role of lateral acquisition in the evolution of these pathogens.

Horizontal gene transference and hybridization play important roles in phytopathogens’ adaptation to new hosts or their acquisition of new mechanisms of virulence [100,101]. A comparative genome analysis of 31 *Phytophthora* species identified genes potentially acquired by horizontal transfer from distantly related species, with homologs associated with pathogenicity, virulence and effector genes from the Pathogen–Host Interaction database (PHI-base) [102]. Closely related species can also exchange genetic material by mobile elements or anastomosis [103,104]. Analysis of the mitogenome of *Ceratocystis huliohia* and *Ceratocystis lukuohia*, which are sexually incompatible co-occurring pathogens in sapwood, evidenced that the regions originated in *C. huliohia* and are actively moving to populations of *C. lukuohia* [105]. Considering the occurrence of many diseases in *T. cacao* crops in the same region such as WBD, BPR and CWT in South American countries, analyzing the evidence of horizontal gene transfer among *T. cacao* pathogens or their ancestors should be an issue studied in future research.

In the initial phase of WBD, *M. perniciosa* secretes many effector proteins [22,69], but only some of them, such as NEP and CP families, have well-understood functions in the current literature. More than 150 putative effectors have been identified in *M. perniciosa* whole-genome sequencing and annotation from *T. cacao* isolates. More than 200 putative effectors have been identified from the closely related species *M. roreri* [14]. Many of those putative effectors should be targeted in future research to provide a better understanding of their function in the pathogenicity of these species in *T. cacao*. Some genes associated with pathogenicity identified only by gene annotation require confirmations in vitro, in planta, and if possible, in field experiments. There are many possible regulations from genes to proteins, and some species have families of NEPs, including pseudogenes. Moreover, experiments with *M. roreri* under culture conditions have already shown conflicting results compared to field conditions [72].

*Ceratocystis cacaofunesta* whole-genome sequencing and annotation [18] also helped predict a considerable number of putative, secreted proteins (342) and confirmed the accumulation of 86 (24%) of them in enriched *T. cacao* xylem culture media. The expansion of the PI-PLC family in the *C. cacaofunesta* genome and its possible role in overcoming the plant initial immune response in susceptible *T. cacao* genotypes has already been discussed in the current literature, but research is lacking on other *C. cacaofunesta* effectors, their molecular functions and their structural characterization.

## 5. Conclusions

This work provides a compilation of important studies on the molecular aspects of *T. cacao* pathogens over the last two decades. The main contribution is an integrated discussion on the recently studied pathogens regarding the knowledge production, distribution and available information about these pathogens.

Research from Brazil and the USA is primarily responsible for most publications about *T. cacao pathogens*, and *M. perniciosa* is the species of cocoa pathogens with the most studies, especially those that involved isolates from cocoa. This is true in terms of the diversity of studies and available data. While some *M. perniciosa* proteins already had been targeted for heterologous expression for characterization, some species have yet to be studied proteomically. Despite the number of works on *M. perniciosa* and witches’ broom disease, there is not, currently, high-quality genome sequence and assembly data publicly available for *M. perniciosa*.

In summary, NEP-like protein coding genes are present in almost all *T. cacao* genomes, and sometimes as gene families, but their effective expression and existence in functional form needs to be confirmed by further experiments. For many *T. cacao* pathogens, the genome size and number of genes appear to be considerably variable, even among different isolates from the same species. In this context, new research involving genome sequencing, transcriptomics and proteomics under plant–pathogen conditions could provide a better understanding of within- and among-species diversity.

## Figures and Tables

**Table 1:** Systematic review research questions.

Questions
What genera and species of eukaryotic microorganism pathogens of *T. cacao* have been the focus of omics studies in recent years?
Which molecular techniques have been applied, and what kind of data are available for each of these species?
How is scientific production about this subject distributed globally?
Which scientific journals and subject areas contain the majority of available studies?
How are the most cited papers found in the systematic review related to each other?
Which proteins for each species are already associated with pathogenicity in the current literature?
Do these proteins have orthologs among the *T. cacao* pathogens discussed in the systematic review?

**Table 2:** Search strings for each scientific database’s advanced search tool.

Database	String
Scopus	TITLE-ABS-KEY (cocoa OR cacao AND pathogen* OR disease*) AND PUBYEAR > 1999 AND (LIMIT-TO (DOCTYPE, “ar”)) AND (LIMIT-TO (LANGUAGE, “English”))
Web of science	TS = (cocoa OR cacao) AND TS = (pathogen* OR disease*) AND Language: (English) AND DOCUMENT TYPE: (Article)

**Table 3:** Whole-genome sequencing studies and data availability.

Paper	Species	Assembly Deposit	Genome Size (Mb)	Number of Gene Models
[13]	*M. perniciosa*	Genbank: GCA_000183025.1	26.7	16,329
[15]	*M. roreri*	Genbank: GCA_000488995.1	52.2	17,910
[16]	*P. megakarya*	Genbank: GCA_002215365.1	101.18	42,036
	*P. palmivora*	Genbank: GCA_002911725.1	107.42	44,327
[18]	*C. cacaofunesta*	Genbank: GCA_002776505.1	30.5	7382
[14]	*M. perniciosa* (MP 4145) *^+^*	http://nbcgib.uesc.br/mperniciosa (accessed on 27 April 2023)	47.01	14,210
	*M. roreri*		45.17	14,154
[74]	*C. theobromae*	Genbank: GCA_009078325.1	31.2	9264
[17]	*P. megakarya*	http://www.cacaopathogenomics.com/ (accessed on 27 April 2023)	222.04 ± 25.19 *	57,577 ± 7904 *
	*P. palmivora*		135.32 ± 17.21 *	36,778 ± 4481 *

^+^ Ref. [14] presents five *M. perniciosa* genomes from different hosts. Mp 4145 was isolated from a susceptible cacao genotype. * Ref. [17] analyzed *P. megakarya* and *P. palmivora* isolate genomes from infected *T. cacao* trees in different countries. Values with an * (asterisk) are in the form mean ± standard deviation.

## Data Availability

No new data were created or analyzed in this study. Data sharing is not applicable to this article.

## Supplementary Materials

The following supporting information can be downloaded at: https://www.mdpi.com/article/10.3390/microorganisms11061567/s1, Figure S1: Word clouds from clusters of papers by topic modeling on abstracts; Table S1: Systematic review protocol.

## References

[1] Beg M.S., Ahmad S., Jan K., Bashir K. (2017). Status, Supply Chain and Processing of Cocoa—A Review. Trends Food Sci. Technol..

[2] Ploetz R.C. (2007). Cacao Diseases: Important Threats to Chocolate Production Worldwide. Phytopathology.

[3] Fulton R.H. (1989). The Cacao Disease Trilogy: Black Pod, Monilia Pod Rot, and Witches’-Broom. Plant Dis..

[4] Evans H.C. (2007). Cacao Diseases—The Trilogy Revisited. Phytopathology.

[5] International Cocoa Organization (ICCO) Pests & Diseases. https://www.icco.org/pests-diseases/.

[6] Puig A.S., Ali S., Strem M., Sicher R., Gutierrez O.A., Bailey B.A. (2018). The Differential Influence of Temperature on *Phytophthora megakarya* and *Phytophthora palmivora* Pod Lesion Expansion, Mycelia Growth, Gene Expression, and Metabolite Profiles. Physiol. Mol. Plant Pathol..

[7] Ali S.S., Amoako-Attah I., Bailey R.A., Strem M.D., Schmidt M., Akrofi A.Y., Surujdeo-Maharaj S., Kolawole O.O., Begoude B.A.D., ten Hoopen G.M. (2016). PCR-Based Identification of Cacao Black Pod Causal Agents and Identification of Biological Factors Possibly Contributing to *Phytophthora megakarya*’s Field Dominance in West Africa. Plant Pathol..

[8] Konam J.K., Guest D.I. (2002). Leaf Litter Mulch Reduces the Survival of *Phytophthora palmivora* under Cocoa Trees in Papua New Guinea. Australas. Plant Pathol..

[9] Torres-de-la-Cruz M., Quevedo-Damián I., Ortiz-García C.F., del Carmen Lagúnez-Espinoza L., Nieto-Angel D., Pérez-de la Cruz M. (2019). Control Químico de Moniliophthora Roreri En México. Biotecnia.

[10] Gutiérrez O.A., Puig A.S., Phillips-Mora W., Bailey B.A., Ali S.S., Mockaitis K., Schnell R.J., Livingstone D., Mustiga G., Royaert S. (2021). SNP Markers Associated with Resistance to Frosty Pod and Black Pod Rot Diseases in an F1 Population of *Theobroma cacao* L. Tree Genet. Genomes.

[11] Montezano Fernandes F., Vieira de Queiroz M., Lopes da Silva L., Maria Queiroz Azevedo D., Luis Badel J., Couto Alfenas A. (2022). Chromosomal Polymorphism of the *Ceratocystis fimbriata* Species Complex in Brazil. Fungal Genet. Biol..

[12] Brown J.S., Phillips-Mora W., Power E.J., Krol C., Cervantes-Martinez C., Motamayor J.C., Schnell R.J. (2007). Mapping QTLs for Resistance to Frosty Pod and Black Pod Diseases and Horticultural Traits in *Theobroma cacao* L. Crop Sci..

[13] Mondego J.M., Carazzolle M.F., Costa G.G., Formighieri E.F., Parizzi L.P., Rincones J., Cotomacci C., Carraro D.M., Cunha A.F., Carrer H. (2008). A Genome Survey of *Moniliophthora perniciosa* Gives New Insights into Witches’ Broom Disease of Cacao. BMC Genom..

[14] Barbosa C.S., da Fonseca R.R., Batista T.M., Barreto M.A., Argolo C.S., de Carvalho M.R., do Amaral D.O.J., de Andrade Silva E.M., Arévalo-Gardini E., Hidalgo K.S. (2018). Genome Sequence and Effectorome of *Moniliophthora perniciosa* and *Moniliophthora roreri* Subpopulations. BMC Genom..

[15] Meinhardt L.W., Costa G.G., Thomazella D.P., Teixeira P.J.P., Carazzolle M., Schuster S.C., Carlson J.E., Guiltinan M.J., Mieczkowski P., Farmer A. (2014). Genome and Secretome Analysis of the Hemibiotrophic Fungal Pathogen, *Moniliophthora roreri*, Which Causes Frosty Pod Rot Disease of Cacao: Mechanisms of the Biotrophic and Necrotrophic Phases. BMC Genom..

[16] Ali S.S., Shao J., Lary D.J., Strem M.D., Meinhardt L.W., Bailey B.A. (2017). *Phytophthora megakarya* and *P. palmivora*, Causal Agents of Black Pod Rot, Induce Similar Plant Defense Responses Late during Infection of Susceptible Cacao Pods. Front. Plant Sci..

[17] Morales-Cruz A., Ali S.S., Minio A., Figueroa-Balderas R., García J.F., Kasuga T., Puig A.S., Marelli J.-P., Bailey B.A., Cantu D. (2020). Independent Whole-Genome Duplications Define the Architecture of the Genomes of the Devastating West African Cacao Black Pod Pathogen *Phytophthora megakarya* and Its Close Relative *Phytophthora palmivora*. G3 Genes Genomes Genet..

[18] Molano E.P.L., Cabrera O.G., Jose J., do Nascimento L.C., Carazzolle M.F., Teixeira P.J.P.L., Alvarez J.C., Tiburcio R.A., Tokimatu Filho P.M., de Lima G.M.A. (2018). *Ceratocystis cacaofunesta* Genome Analysis Reveals a Large Expansion of Extracellular Phosphatidylinositol-Specific Phospholipase-C Genes (PI-PLC). BMC Genom..

[19] Garcia O., Macedo J.A.N., Tibúrcio R., Zaparoli G., Rincones J., Bittencourt L.M.C., Ceita G.O., Micheli F., Gesteira A., Mariano A.C. (2007). Characterization of Necrosis and Ethylene-Inducing Proteins (NEP) in the Basidiomycete *Moniliophthora perniciosa*, the Causal Agent of Witches’ Broom in *Theobroma cacao*. Mycol. Res..

[20] Bae H., Bowers J.H., Tooley P.W., Bailey B.A. (2005). NEP1 Orthologs Encoding Necrosis and Ethylene Inducing Proteins Exist as a Multigene Family in *Phytophthora megakarya*, Causal Agent of Black Pod Disease on Cacao. Mycol. Res..

[21] De Oliveira Barsottini M.R., de Oliveira J.F., Adamoski D., Teixeira P.J.P.L., do Prado P.F.V., Tiezzi H.O., Sforça M.L., Cassago A., Portugal R.V., de Oliveira P.S.L. (2013). Functional Diversification of Cerato-Platanins in *Moniliophthora perniciosa* as Seen by Differential Expression and Protein Function Specialization. Mol. Plant Microbe Interact..

[22] Santos A.S., Mora-Ocampo I.Y., de Novais D.P.S., Aguiar E.R.G.R., Pirovani C.P. (2023). State of the Art of the Molecular Biology of the Interaction between Cocoa and Witches’ Broom Disease: A Systematic Review. Int. J. Mol. Sci..

[23] Jiménez D.L., Alvarez J.C., Mosquera S. (2022). Frosty Pod Rot: A Major Threat to Cacao Plantations on the Move. Trop. Plant Pathol..

[24] Marelli J.-P., Guest D.I., Bailey B.A., Evans H.C., Brown J.K., Junaid M., Barreto R.W., Lisboa D.O., Puig A.S. (2019). Chocolate Under Threat from Old and New Cacao Diseases. Phytopathology.

[25] Page M.J., McKenzie J.E., Bossuyt P.M., Boutron I., Hoffmann T.C., Mulrow C.D., Shamseer L., Tetzlaff J.M., Akl E.A., Brennan S.E. (2021). The PRISMA 2020 Statement: An Updated Guideline for Reporting Systematic Reviews. BMJ.

[26] Fabbri S., Silva C., Hernandes E., Octaviano F., Di Thommazo A., Belgamo A. Improvements in the StArt Tool to Better Support the Systematic Review Process. Proceedings of the 20th International Conference on Evaluation and Assessment in Software Engineering.

[27] (2023). R Core Team R: A Language and Environment for Statistical Computing.

[28] Blei D.M. (2003). Latent Dirichlet Allocation. J. Mach. Learn. Res..

[29] Jones T. TextmineR: Functions for Text Mining and Topic Modeling. https://CRAN.R-project.org/package=textmineR.

[30] Nikita M. Ldatuning: Tuning of the Latent Dirichlet Allocation Models Parameters. https://CRAN.R-project.org/package=ldatuning.

[31] Frey B.J., Dueck D. (2007). Clustering by Passing Messages Between Data Points. Science.

[32] Bodenhofer U., Kothmeier A., Hochreiter S. (2011). APCluster: An R Package for Affinity Propagation Clustering. Bioinformatics.

[33] Charrad M., Ghazzali N., Boiteau V., Niknafs A. (2014). NbClust: An R Package for Determining the Relevant Number of Clusters in a Data Set. J. Stat. Softw..

[34] Fellows I. Wordcloud Makes Words Less Cloudy. https://blog.fellstat.com/?p=248.

[35] Müller K., Ooms J., James D., DebRoy S., Wickham H., Horner J. RMariaDB: Database Interface and MariaDB Driver. https://CRAN.R-project.org/package=RMariaDB.

[36] Wickham H. (2016). Ggplot2: Elegant Graphics for Data Analysis.

[37] Aria M., Cuccurullo C. (2017). Bibliometrix: An R-Tool for Comprehensive Science Mapping Analysis. J. Informetr..

[38] Van Eck N.J., Waltman L., Ding Y., Rousseau R., Wolfram D. (2014). Visualizing Bibliometric Networks. Measuring Scholarly Impact.

[39] Sayers E.W., Bolton E.E., Brister J.R., Canese K., Chan J., Comeau D.C., Connor R., Funk K., Kelly C., Kim S. (2022). Database Resources of the National Center for Biotechnology Information. Nucleic Acids Res..

[40] Altschul S.F., Gish W., Miller W., Myers E.W., Lipman D.J. (1990). Basic Local Alignment Search Tool. J. Mol. Biol..

[41] Xu L., Dong Z., Fang L., Luo Y., Wei Z., Guo H., Zhang G., Gu Y.Q., Coleman-Derr D., Xia Q. (2019). OrthoVenn2: A Web Server for Whole-Genome Comparison and Annotation of Orthologous Clusters across Multiple Species. Nucleic Acids Res..

[42] Bailey T.L., Elkan C. (1994). Fitting a Mixture Model by Expectation Maximization to Discover Motifs in Bipolymers. Proc. Int. Conf. Intell. Syst. Mol. Biol..

[43] Bailey T.L., Boden M., Buske F.A., Frith M., Grant C.E., Clementi L., Ren J., Li W.W., Noble W.S. (2009). MEME Suite: Tools for Motif Discovery and Searching. Nucleic Acids Res..

[44] Ali S.S., Shao J., Strem M.D., Phillips-Mora W., Zhang D., Meinhardt L.W., Bailey B.A. (2015). Combination of RNAseq and SNP Nanofluidic Array Reveals the Center of Genetic Diversity of Cacao Pathogen *Moniliophthora roreri* in the Upper Magdalena Valley of Colombia and Its Clonality. Front. Microbiol..

[45] Jaimes Y.Y., Gonzalez C., Rojas J., Cornejo O.E., Mideros M.F., Restrepo S., Cilas C., Furtado E.L. (2016). Geographic Differentiation and Population Genetic Structure of *Moniliophthora roreri* in the Principal Cocoa Production Areas in Colombia. Plant Dis..

[46] Maridueña-Zavala M.G., Freire-Peñaherrera A., Espinoza-Lozano R.F., Villavicencio-Vasquez M., Jimenez-Feijoo M., Cevallos-Cevallos J.M. (2019). Genetic Characterization of *Moniliophthora perniciosa* from Ecuador and in Vitro Sensitivity to Compost Tea. Eur. J. Plant Pathol..

[47] Brugman E., Wibowo A., Widiastuti A. (2022). *Phytophthora palmivora* from Sulawesi and Java Islands, Indonesia, Reveals High Genotypic Diversity and Lack of Population Structure. Fungal Biol..

[48] Veerappa Hanumanthappa P., Hegde V., Kuriyathadka Mahalingeshwara S., Muliyar Krishna R., Kaitheri Edathil R., Pallem C. (2018). Differentiation of Phytophthora Species Associated with Plantation Crops Using PCR and High-Resolution Melting Curve Analysis. J. Plant Pathol..

[49] De Oliveira B.V., Teixeira G.S., Reis O., Barau J.G., Teixeira P.J.P.L., do Rio M.C.S., Domingues R.R., Meinhardt L.W., Paes Leme A.F., Rincones J. (2012). A Potential Role for an Extracellular Methanol Oxidase Secreted by *Moniliophthora perniciosa* in Witches’ Broom Disease in Cacao. Fungal Genet. Biol..

[50] Pungartnik C., Melo S.C.O., Basso T.S., Macena W.G., Cascardo J.C.M., Brendel M. (2009). Reactive Oxygen Species and Autophagy Play a Role in Survival and Differentiation of the Phytopathogen *Moniliophthora perniciosa*. Fungal Genet. Biol..

[51] Santana L.S., Costa M.G.C., Pirovani N.M., Almeida A.F., Alvim F.C., Pirovani C.P. (2014). TcCYS4, a Cystatin from Cocoa, Reduces Necrosis Triggered by MpNEP2 in Tobacco Plants. Genet. Mol. Res..

[52] Artero A.S., Silva J.Q., Albuquerque P.S.B., Bressan E.A., Leal G.A., Sebbenn A.M., Griffith G.W., Figueira A. (2017). Spatial Genetic Structure and Dispersal of the Cacao Pathogen *Moniliophthora perniciosa* in the Brazilian Amazon. Plant Pathol..

[53] Gramacho K.P., Risterucci A.M., Lanaud C., Lima L.S., Lopes U.V. (2006). Characterization of Microsatellites in the Fungal Plant Pathogen Crinipellis Perniciosa: PRIMER NOTE. Mol. Ecol. Notes.

[54] Rincones J., Mazotti G.D., Griffith G.W., Pomela A., Figueira A., Leal G.A., Queiroz M.V., Pereira J.F., Azevedo R.A., Pereira G.A.G. (2006). Genetic Variability and Chromosome-Length Polymorphisms of the Witches’ Broom Pathogen *Crinipellis perniciosa* from Various Plant Hosts in South America. Mycol. Res..

[55] Mares J.H., Gramacho K.P., Santana J.O., Oliveira de Souza A., Alvim F.C., Pirovani C.P. (2020). Hydrosoluble Phylloplane Components of *Theobroma cacao* Modulate the Metabolism of Moniliophthora Perniciosa Spores during Germination. Fungal Biol..

[56] Santos Junior M.C., Gonçalves P.A., Taranto A.G., Koblitz M.G.B., Góes-Neto A., Pirovani C.P., Cascardo J.C.M., da Cruz S.H., Zingali R.B., Pereira G.A.G. (2011). Purification, Characterization and Structural Determination of UDP-N-Acetylglucosamine Pyrophosphorylase Produced by *Moniliophthora perniciosa*. J. Braz. Chem. Soc..

[57] Teixeira P.J.P.L., Thomazella D.P.T., Vidal R.O., do Prado P.F.V., Reis O., Baroni R.M., Franco S.F., Mieczkowski P., Pereira G.A.G., Mondego J.M.C. (2012). The Fungal Pathogen *Moniliophthora perniciosa* Has Genes Similar to Plant PR-1 That Are Highly Expressed during Its Interaction with Cacao. PLoS ONE.

[58] Bailey B.A., Crozier J., Sicher R.C., Strem M.D., Melnick R., Carazzolle M.F., Costa G.G.L., Pereira G.A.G., Zhang D., Maximova S. (2013). Dynamic Changes in Pod and Fungal Physiology Associated with the Shift from Biotrophy to Necrotrophy during the Infection of *Theobroma cacao* by *Moniliophthora roreri*. Physiol. Mol. Plant Pathol..

[59] Melnick R.L., Strem M.D., Crozier J., Sicher R.C., Bailey B.A. (2013). Molecular and Metabolic Changes of Cherelle Wilt of Cacao and Its Effect on *Moniliophthora roreri*. Physiol. Mol. Plant Pathol..

[60] Aime M.C., Phillips-Mora W. (2005). The Causal Agents of Witches’ Broom and Frosty Pod Rot of Cacao (Chocolate, *Theobroma cacao*) Form a New Lineage of Marasmiaceae. Mycologia.

[61] Engelbrecht C.J.B., Harrington T.C. (2005). Intersterility, Morphology and Taxonomy of *Ceratocystis fimbriata* on Sweet Potato, Cacao and Sycamore. Mycologia.

[62] Decloquement J., Ramos-Sobrinho R., Elias S.G., Britto D.S., Puig A.S., Reis A., da Silva R.A.F., Honorato-Júnior J., Martins Newman Luz E.D., Pinho D.B. (2021). *Phytophthora theobromicola* sp. Nov.: A New Species Causing Black Pod Disease on Cacao in Brazil. Front. Microbiol..

[63] Santana M.F., de Araújo E.F., de Souza J.T., Mizubuti E.S.G., de Queiroz M.V. (2012). Development of Molecular Markers Based on Retrotransposons for the Analysis of Genetic Variability in *Moniliophthora perniciosa*. Eur. J. Plant Pathol..

[64] Silva J.R.Q., Figueira A., Pereira G.A.G., Albuquerque P. (2008). Development of Novel Microsatellites from *Moniliophthora perniciosa*, Causal Agent of the Witches’ Broom Disease of *Theobroma cacao*: Permanent genetic resources. Mol. Ecol. Resour..

[65] Melo B.L.B., de Souza J.T., Santos R.M.F., Rehner S.A., Solis K.H., Suarez C., Hebbar P.K., Lemos L.S.L., Gramacho K.P. (2014). Development of Microsatellites for the Cacao Frosty Pod Rot Pathogen, *Moniliophthora roreri*. For. Pathol..

[66] Chowdappa P., Brayford D., Smith J., Flood J. (2003). Molecular Discrimination of Phytophthora Isolates on Cocoa and Their Relationship with Coconut, Black Pepper and Bell Pepper Isolates Based on RDNA Repeat and AFLP Fingerprints. Curr. Sci..

[67] Guha Roy S., Bhattacharyya S., Mukherjee S.K., Khatua D.C. (2009). Molecular Identification of *Phytophthora* spp. Affecting Some Economically Important Crops in Eastern India through ITS-RFLP and Sequencing of the ITS Region: *Phytophthora* spp. Affecting Crops in Eastern India. J. Phytopathol..

[68] Mfegue C.V., Herail C., Adreit H., Mbenoun M., Techou Z., Ten Hoopen M., Tharreau D., Ducamp M. (2012). Microsatellite Markers for Population Studies of *Phytophthora megakarya* (Pythiaceae), a Cacao Pathogen in Africa. Am. J. Bot..

[69] Rincones J., Scarpari L.M., Carazzolle M.F., Mondego J.M.C., Formighieri E.F., Barau J.G., Costa G.G.L., Carraro D.M., Brentani H.P., Vilas-Boas L.A. (2008). Differential Gene Expression Between the Biotrophic-Like and Saprotrophic Mycelia of the Witches’ Broom Pathogen *Moniliophthora perniciosa*. Mol. Plant Microbe Interact.

[70] De Freitas Franco S., Baroni R.M., Carazzolle M.F., Teixeira P.J.P.L., Reis O., Pereira G.A.G., Mondego J.M.C. (2015). Genomic Analyses and Expression Evaluation of Thaumatin-like Gene Family in the Cacao Fungal Pathogen *Moniliophthora perniciosa*. Biochem. Biophys. Res. Commun..

[71] Bailey B.A., Melnick R.L., Strem M.D., Crozier J., Shao J., Sicher R., Phillips-Mora W., Ali S.S., Zhang D., Meinhardt L. (2014). Differential Gene Expression by *Moniliophthora roreri* While Overcoming Cacao Tolerance in the Field: The *M. roreri* Interaction with Tolerant Cacao. Mol. Plant Pathol..

[72] Bailey B.A., Ali S.S., Strem M.D., Meinhardt L.W. (2018). Morphological Variants of *Moniliophthora roreri* on Artificial Media and the Biotroph/Necrotroph Shift. Fungal Biol..

[73] Masanto M., Wibowo A., Ridwan N.F., Sawitri W.D., Kageyama K., Subandiyah S. (2021). The Expression of Pathogenicity-Related Genes in *Phytophthora palmivora* Causing Black Pod Rot Disease on Cacao (*Theobroma cacao* L.) in Indonesia. J. Plant Interact..

[74] Ali S.S., Asman A., Shao J., Firmansyah A.P., Susilo A.W., Rosmana A., McMahon P., Junaid M., Guest D., Kheng T.Y. (2019). Draft Genome Sequence of Fastidious Pathogen *Ceratobasidium theobromae*, Which Causes Vascular-Streak Dieback in Theobroma Cacao. Fungal Biol. Biotechnol..

[75] Santos Gomes D., de Andrade Silva E.M., de Andrade Rosa E.C., Silva Gualberto N.G., de Jesus Souza M.Á., Santos G., Pirovani C.P., Micheli F. (2021). Identification of a Key Protein Set Involved in *Moniliophthora perniciosa* Necrotrophic Mycelium and Basidiocarp Development. Fungal Genet. Biol..

[76] Silva F.A.C., Pirovani C.P., Menezes S., Pungartnik C., Santiago A.S., Costa M.G.C., Micheli F., Gesteira A.S. (2013). Proteomic Response of *Moniliophthora perniciosa* Exposed to Pathogenesis-Related Protein-10 from *Theobroma cacao*. Genet. Mol. Res..

[77] Zaparoli G., de Oliveira Barsottini M.R., de Oliveira J.F., Dyszy F., Teixeira P.J.P.L., Barau J.G., Garcia O., Costa-Filho A.J., Ambrosio A.L.B., Pereira G.A.G. (2011). The Crystal Structure of Necrosis- and Ethylene-Inducing Protein 2 from the Causal Agent of Cacao’s Witches’ Broom Disease Reveals Key Elements for Its Activity. Biochemistry.

[78] Baroni R.M., Luo Z., Darwiche R., Hudspeth E.M., Schneiter R., Pereira G.A.G., Mondego J.M.C., Asojo O.A. (2017). Crystal Structure of MpPR-1i, a SCP/TAPS Protein from *Moniliophthora perniciosa*, the Fungus That Causes Witches’ Broom Disease of Cacao. Sci. Rep..

[79] Zaparoli G., Cabrera O.G., Medrano F.J., Tiburcio R., Lacerda G., Pereira G.G. (2009). Identification of a Second Family of Genes in *Moniliophthora perniciosa*, the Causal Agent of Witches’ Broom Disease in Cacao, Encoding Necrosis-Inducing Proteins Similar to Cerato-Platanins. Mycol. Res..

[80] Teh C.-Y., Pang C.-L., Tor X.-Y., Ho P.-Y., Lim Y.-Y., Namasivayam P., Ho C.-L. (2019). Molecular Cloning and Functional Analysis of a Necrosis and Ethylene Inducing Protein (NEP) from Ganoderma Boninense. Physiol. Mol. Plant Pathol..

[81] Golinelli D., Nuzzolese A.G., Sanmarchi F., Bulla L., Mongiovì M., Gangemi A., Rucci P. (2022). Semi-Automatic Systematic Literature Reviews and Information Extraction of COVID-19 Scientific Evidence: Description and Preliminary Results of the COKE Project. Information.

[82] Yu Z., Carver J.C., Rothermel G., Menzies T. (2022). Assessing Expert System-Assisted Literature Reviews with a Case Study. Expert Syst. Appl..

[83] Food and Agriculture Organization of the United Nations (FAO) Crops and Livestock Products. https://www.fao.org/faostat/en/#data/QCL.

[84] Pereira J.L., de Almeida L.C.C., Santos S.M. (1996). Witches’ Broom Disease of Cocoa in Bahia: Attempts at Eradication and Containment. Crop Prot..

[85] Ekdale B., Rinaldi A., Ashfaquzzaman M., Khanjani M., Matanji F., Stoldt R., Tully M. (2022). Geographic Disparities in Knowledge Production: A Big Data Analysis of Peer-Reviewed Communication Publications from 1990 to 2019. Int. J. Commun..

[86] SCIMAGO Scimago Journal & Country Rank. https://www.scimagojr.com/countryrank.php.

[87] Paladines-Rezabala A., Moreira-Morrillo A.A., Mieles A.E., Garcés-Fiallos F.R. (2022). Advances in Understanding of the Interaction between *Ceratocystis cacaofunesta* and Xyleborus Ferrugineus (Coleoptera: Curculionidae: Scolytinae) on Cocoa Trees. Sci. Agropecu..

[88] Ramos-Sobrinho R., Chingandu N., Gutierrez O.A., Marelli J.P., Brown J.K. (2020). A Complex of Badnavirus Species Infecting Cacao Reveals Mixed Infections, Extensive Genomic Variability, and Interspecific Recombination. Viruses.

[89] Mora-Ocampo I.Y., Pirovani C.P., Luz E.D.M.N., Rêgo A.P.B., Silva E.M.A., Rhodes-Valbuena M., Corrêa R.X. (2021). *Ceratocystis cacaofunesta* Differentially Modulates the Proteome in Xylem-Enriched Tissue of Cocoa Genotypes with Contrasting Resistance to Ceratocystis Wilt. Planta.

[90] Mucherino Muñoz J.J., De Melo C.A.F., Santana Silva R.J., Luz E.D.M.N., Corrêa R.X. (2021). Structural and Functional Genomics of the Resistance of Cacao to *Phytophthora palmivora*. Pathogens.

[91] Barreto M.A., Rosa J.R.B.F., Holanda I.S.A., Cardoso-Silva C.B., Vildoso C.I.A., Ahnert D., Souza M.M., Corrêa R.X., Royaert S., Marelli J. (2018). QTL Mapping and Identification of Corresponding Genomic Regions for Black Pod Disease Resistance to Three *Phytophthora* Species in *Theobroma cacao* L. Euphytica.

[92] Albuquerque P.S.B. (2006). Mapas de Ligação e Identificação de Locos Controladores de Características Quantitativas (QTLs) Associados à Resistência a Crinipellis perniciosa Em Acessos de Cacaueiro (Theobroma cacao) Originários da Amazônia Brasileira.

[93] Jaimez R.E., Barragan L., Fernández-Niño M., Wessjohann L.A., Cedeño-Garcia G., Sotomayor Cantos I., Arteaga F. (2022). *Theobroma cacao* L. Cultivar CCN 51: A Comprehensive Review on Origin, Genetics, Sensory Properties, Production Dynamics, and Physiological Aspects. PeerJ.

[94] De Mello Paim V.R.L., Luz E.D.M.N., Pires J.L., Silva S.D.V.M., de Souza J.T., Albuquerque P.S.B., Santos Filho L.P. (2006). dos Sources of Resistance to *Crinipellis perniciosa* in Progenies of Cacao Accessions Collected in the Brazilian Amazon. Sci. Agric..

[95] Tirado-Gallego P.A., Lopera-Álvarez A., Ríos-Osorio L.A. (2016). Estrategias de Control de *Moniliophthora roreri* y *Moniliophthora perniciosa* en *Theobroma cacao* L.: Revisión Sistemática. Cienc. Tecnol. Agropecu..

[96] Krauss U., Hidalgo E., Bateman R., Adonijah V., Arroyo C., García J., Crozier J., Brown N.A., ten Hoopen G.M., Holmes K.A. (2010). Improving the Formulation and Timing of Application of Endophytic Biocontrol and Chemical Agents against Frosty Pod Rot (*Moniliophthora roreri*) in Cocoa (*Theobroma cacao*). Biol. Control.

[97] Loguercio L.L., de Carvalho A.C., Niella G.R., De Souza J.T., Pomella A.W.V. (2009). Selection of *Trichoderma stromaticum* Isolates for Efficient Biological Control of Witches’ Broom Disease in Cacao. Biol. Control.

[98] Bertier L., Leus L., D’hondt L., de Cock A.W.A.M., Höfte M. (2013). Host Adaptation and Speciation through Hybridization and Polyploidy in Phytophthora. PLoS ONE.

[99] Tiburcio R.A., Costa G.G.L., Carazzolle M.F., Mondego J.M.C., Schuster S.C., Carlson J.E., Guiltinan M.J., Bailey B.A., Mieczkowski P., Meinhardt L.W. (2010). Genes Acquired by Horizontal Transfer Are Potentially Involved in the Evolution of Phytopathogenicity in *Moniliophthora perniciosa* and *Moniliophthora roreri*, Two of the Major Pathogens of Cacao. J. Mol. Evol..

[100] Vries S., Stukenbrock E.H., Rose L.E. (2020). Rapid Evolution in Plant–Microbe Interactions—An Evolutionary Genomics Perspective. New Phytol..

[101] Wang J., Xiao S., Zheng L., Pan Y., Zhao D., Zhang D., Li Q., Zhu J., Yang Z. (2022). Multiomic Approaches Reveal Novel Lineage-Specific Effectors in the Potato and Tomato Early Blight Pathogen Alternaria Solani. Phytopathol. Res..

[102] Kronmiller B.A., Feau N., Shen D., Tabima J.F., Ali S.S., Armitage A.D., Arredondo F., Bailey B.A., Bollmann S.R., Dale A. (2023). Comparative Genomic Analysis of 31 *Phytophthora* Genomes Reveals Genome Plasticity and Horizontal Gene Transfer. Mol. Plant-Microbe Interact..

[103] Soanes D., Richards T.A. (2014). Horizontal Gene Transfer in Eukaryotic Plant Pathogens. Annu. Rev. Phytopathol..

[104] Mehrabi R., Bahkali A.H., Abd-Elsalam K.A., Moslem M., Ben M’Barek S., Gohari A.M., Jashni M.K., Stergiopoulos I., Kema G.H.J., de Wit P.J.G.M. (2011). Horizontal Gene and Chromosome Transfer in Plant Pathogenic Fungi Affecting Host Range. FEMS Microbiol. Rev..

[105] Mayers C.G., Harrington T.C., Wai A., Hausner G. (2021). Recent and Ongoing Horizontal Transfer of Mitochondrial Introns Between Two Fungal Tree Pathogens. Front. Microbiol..

