# Supplementary material for: Genomic and Pathogenicity Mechanisms of the Main Theobroma cacao L. Eukaryotic Pathogens: A Systematic Review
Source: Microorganisms. 2023 Jun 13;11(6):1567. doi: 10.3390/microorganisms11061567 (PMC10304304; doi:10.3390/microorganisms11061567)
Supplement: Supplementary file 1 [file microorganisms-11-01567-s001.zip › Table S1_Systematic_Review_Protocol.pdf]

Molecular knowledge about *Theobroma cacao* L. eukaryotic pathogens: a systematic review

1- Systematic Review Protocol

**General information**	
Title	**Molecular knowledge about *Theobroma cacao* L. eukaryotic pathogens: a systematic review**
Researchers	Diogo Pereira Silva de Novais<br>Thiago Mafra Batista<br>Eduardo Almeida Costa<br>Carlos Priminho Pirovani
Description	A set of diseases caused by fungi and oomycetes are responsible for large losses in annual world cocoa production. Managing the impact caused by these diseases is very complex because a common solution must be found for different pathogens. In this context, systematic knowledge of *Theobroma cacao* L. pathogens' molecular characteristics may help researchers understand the possibilities and limitations of cocoa disease management strategies. This work systematically organizes and summarizes the main findings of omic studies of *T. cacao* eukaryotic pathogens, focusing on the plant-pathogen interaction and production dynamics.
Objectives	This research aims to understand the state of the art of molecular studies in eukaryotic cocoa pathogenic microorganisms and what is already known about mechanisms of pathogenicity. It has the following specific objectives:<br>● Identify the main *T. cacao* pathogens discussed in current scientific literature.<br>● Analysis knowledge production on *T. cacao* pathogen fungi and oomycetes genomes and molecular aspects of pathogenicity<br>● Identify genes and proteins associated with pathogenicity in *T. cacao* pathogens.
**Research question**	
Questions	1. What genera and species of eukaryotic microorganism pathogens of *T. cacao* have been the focus of omic studies in recent years?<br>2. Which molecular techniques have been applied, and what kind of data are available for each of these species?

	3. How is scientific production about this subject distributed globally?<br>4. Which scientific journals and subject areas contain the majority of available studies?<br>5. How are the most cited papers found in the systematic review related to each other?<br>6. Which proteins for each species are already associated with pathogenicity in the current literature?<br>7. Do these proteins have orthologs among the *T. cacao* pathogens discussed in the systematic review?
Population	Eukaryotic microorganisms pathogen of *T. cacao*
Intervention	Not applicable
Comparison	- Homologous proteins secreted by different pathogens<br>- Genes shared exclusively between pathogens<br>- Genes or proteins pointed in the literature as associated with the pathogenicity
Hypothesis	There are genes shared among species of *T. cacao* pathogens associated with the pathogenicity. Some of those genes codifies proteins to be secreted.
Expected results	This systematic review aims to understand what are the main cocoa pathogens eukaryotic microorganisms and their known molecular mechanisms of pathogenicity to support the understanding of shared characteristics of cocoa pathogens and provides information for new research to elucidate the origin and mechanisms of pathogenicity in different species.
Types of studies	Primary studies papers
**Study identification**	
Key words	*Moniliophthora*, *Phytophthora*, *Ceratocystis cacaofunesta*, witches' broom disease, black pod disease, *Ceratocystis* wilt, frosty pod rot
Research strings	**Scopus database**<br><br>TITLE-ABS-KEY (cocoa OR cacao AND pathogen* OR disease*) AND PUBYEAR > 1999 AND (LIMIT-TO (DOCTYPE, "ar")) AND (LIMIT-TO (LANGUAGE , "English"))<br><br>**Web of science database**<br><br>TS=(cocoa OR cacao) AND TS=(pathogen* OR disease*) AND Language: (English) AND DOCUMENT TYPE: (Article)
Selection criteria	- Peer reviewed databases
Sources of search	Scopus and Web of Science
Search tool	Direct on database advanced search tools

Studies selection and evaluation	
Inclusion and exclusion criteria	Inclusion:<br>- Written in English;<br>- Published between 2000 and 2022 (June);<br>- Primary studies;<br>- Papers which discusses genome, transcriptome or proteome of *T. cacao* pathogens eukaryotic microorganisms.<br><br>Exclusion:<br>- Papers not related to the objectives of the work;<br>- Research without molecular analyses of pathogens;
Studies initial selection	- Papers will be clustered using the *Affinity Propagation* algorithm, by similarities in title, abstract and keywords. Papers in clusters which the representative is not related to the objectives of this review will be excluded;<br>- Papers will be excluded by reading the title, abstract and keywords;<br>- Papers will be selected reading the full text.
Final selection	- To have all inclusion criteria;<br>- Do not have any exclusion criteria;<br>- Be selected by full text reading.
Studies quality evaluation	● Relation with the systematic review={Low related(1),Related(2),Very related(3)}<br>● Results={Not presented(1),Inconclusive(2),Clear contribution (3)}<br>● Availability of research data={Not available (1),Avaiable with low documentation(2),Available and documented(3)}
**Data extraction and summarization**	
Data extraction form fields	- Title;<br>- Abstract;<br>- Key words;<br>- Pathogen’s genera and species;<br>- Type of molecular analyses;<br>- Genes associated with the pathogenicity<br>- Proteins associated with the pathogenicity
Results summarization	Tables, graphics with univariate and multivariate analyses. The data extracted from the selected papers will be stored in a relational database and SQL searches will be done to summarizes information such as:<br>● Amount of papers for type of molecular analyses;

	• Mean of genes differentially expressed by species of pathogen;<br>• Amount of research by country related to each pathogen;<br>• Mean of proteins secreted by each pathogen.
Publication	Results will be reported as a paper submitted to high quality journal specialized on microorganisms and/or plant pathogens.
