# Supplementary figures and images for: Genomic and Pathogenicity Mechanisms of the Main Theobroma cacao L. Eukaryotic Pathogens: A Systematic Review
Source: Microorganisms. 2023 Jun 13;11(6):1567. doi: 10.3390/microorganisms11061567 (PMC10304304; doi:10.3390/microorganisms11061567)

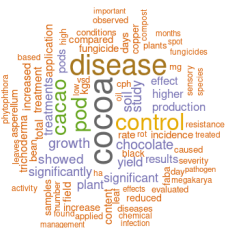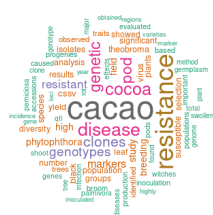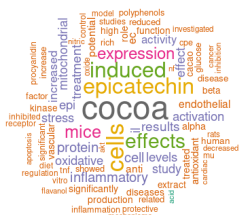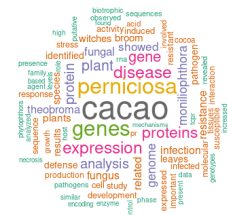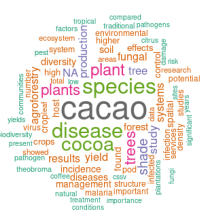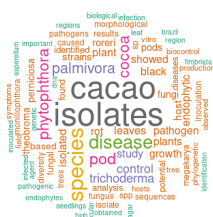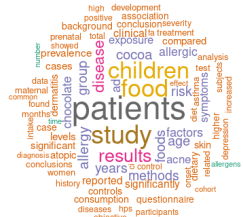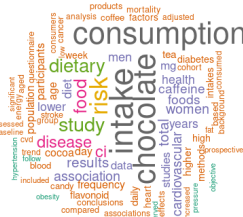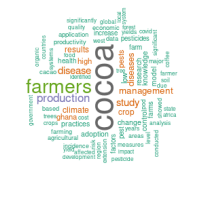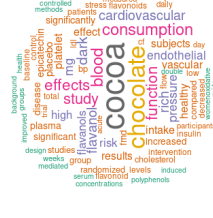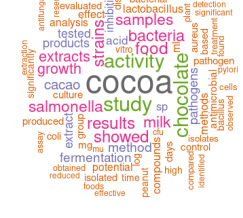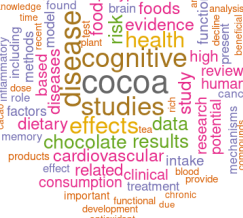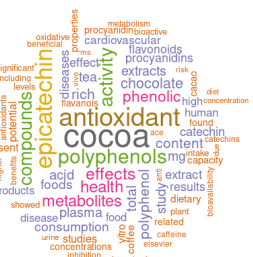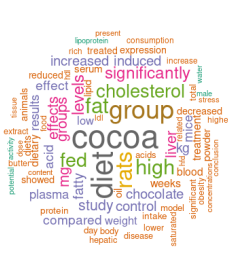

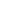 Selected for title and abstract reading

Supplement: Supplementary file 1 [file microorganisms-11-01567-s001.zip › Figure S1_Selected_Clusters.pdf]
